# Adaptive dual-window enhancement and multi-scale texture prior fusion for robust kidney CT classification

**DOI:** 10.1371/journal.pone.0335585

**Published:** 2025-11-07

**Authors:** Ping Xia, Yilin Li, Xin Yao, Yunjia Jiang, WeiMing He, Ming-gang Wei

**Affiliations:** 1 The First Affiliated Hospital of Soochow University, Suzhou, China; 2 Suzhou Traditional Chinese Medicine Hospital affiliated to Nanjing University of Chinese Medicine, Suzhou, China; 3 Affiliated Hospital of Nanjing University of Chinese Medicine, Nanjing, China; Chongqing Normal University, CHINA

## Abstract

Accurate classification of kidney diseases is of great importance for clinical diagnosis and treatment. However, traditional CT images suffer from insufficient contrast, blurred tissue boundaries, and complex texture variations, which limit the performance of automated analysis. This paper proposes a novel kidney CT classification framework that combines Adaptive Dual-Window Enhancement (ADWE) with Multi-Scale Texture Prior Fusion (MTPF). The ADWE module dynamically adjusts window width and window level to generate complementary views, effectively enhancing the contrast of both soft tissues and high-density structures; the MTPF module incorporates edge, local binary pattern (LBP), and Gabor texture priors to achieve fine-grained structural modeling. Experimental results demonstrate that in the binary classification task, the proposed method achieves an accuracy of 0.9802, F1-score of 0.9786, and AUC of 0.9989, all outperforming mainstream deep learning and domain-specific medical models. In the four-class classification task, it achieves an accuracy of 0.8821, F1-score of 0.8438, and AUC of 0.9801, representing an improvement of approximately 3%–5% compared with the ConvNeXtV2 baseline. Moreover, under noise intensity σ=0.1, the method still maintains an accuracy of 0.8510 and an AUC of 0.9634, showing remarkable robustness. These results validate the effectiveness and clinical potential of the proposed method for automated kidney CT classification.

## 1 Introduction

Kidney diseases such as cysts, stones, and tumors have high incidence and severity in clinical practice, and timely and accurate diagnosis is crucial for treatment planning and patient prognosis [1]. Computed tomography (CT) has become a common method for kidney disease detection and classification because it provides high-resolution anatomical information [2,3]. However, the performance of traditional techniques such as fitting Gaussian mixture models and multi-layer perceptron-based methods [4,5] can be limited due to many factors (e.g., similar intensity, artifacts, low contrast in some slices), while manual kidney CT image analysis mainly relies on manual interpretation, which is easily influenced by observer experience and subjective factors. At the same time, although deep learning methods have achieved remarkable progress in medical image classification, existing models still face challenges such as insufficient discriminative power, limited robustness, and weak clinical interpretability due to large contrast variations, blurred tissue boundaries, and complex lesion textures in CT imaging [6,7]. These challenges, to some extent, hinder the widespread adoption and application of automated methods in real-world clinical scenarios.

To address the above issues, this paper proposes a novel kidney CT classification framework that integrates Adaptive Dual-Window Enhancement with Multi-Scale Texture Prior Fusion. The ADWE module adaptively learns window width and window level parameters to generate complementary dual-view images, thereby enhancing the contrast of both soft tissues and high-density structures and alleviating the information loss caused by conventional single-window settings. The MTPF module extracts multi-scale texture priors (including edge features, local binary patterns, and Gabor filter responses) from the enhanced images and fuses them with deep features to capture fine-grained texture and boundary information. These two modules form a complementary relationship at the feature level, improving the discriminative capacity of the model while enhancing its adaptability to noise and complex lesions.

The main contributions of this paper can be summarized as follows:

- A novel Adaptive Dual-Window Enhancement module is proposed, which can dynamically generate multi-view CT images, significantly improving contrast representation in different grayscale regions and enhancing the model’s sensitivity to various lesion types.
- A Multi-Scale Texture Prior Fusion module is designed, which incorporates edge, local binary pattern, and frequency-domain features into the deep learning framework, effectively capturing fine-grained texture differences among cysts, stones, and tumors.
- Extensive experiments are conducted on two public kidney CT datasets, and the results demonstrate that the proposed method outperforms mainstream deep learning models and domain-specific medical approaches in both binary and four-class classification tasks, while exhibiting stronger robustness and clinical applicability under noisy conditions.

## 2 Related work

### 2.1 Medical image classification approaches

With the rapid development of deep learning, medical image classification tasks have achieved significant progress.In addition to traditional convolutional neural network (CNN) frameworks, recent studies have further explored multimodal and application-specific architectures for more accurate and robust medical diagnostics. For instance, Nakach et al. conducted a comprehensive investigation of multimodal fusion strategies for breast cancer classification [8], while Göçeri proposed CNN-based desktop systems for dermatological disease classification and performed comparative analyses of different network variants [9,10]. Similarly, Idlahcen et al. examined data mining and machine learning approaches in gynecologic oncology, highlighting the growing role of AI in diverse clinical contexts [11]. Early studies mainly relied on convolutional neural networks (CNNs) and their variants for the automatic recognition of anatomical structures and organs [12–14]. These methods demonstrated strong performance in feature extraction and representation learning, and were gradually extended to more complex medical scenarios such as multimodal analysis and cross-domain transfer. However, general image classification networks often encounter limitations when dealing with the complex tissue structures and imaging variations in medical images, including insufficient sensitivity to contrast and limited capability in capturing fine-grained textures. Therefore, structural improvements and targeted optimizations are required.

In the field of kidney CT image classification, researchers have proposed a variety of deep learning models to address multi-class diagnostic tasks such as cysts, stones, and tumors. For instance, Sharma et al. introduced a hybrid deep learning framework that combines ResNet101 with a customized CNN to achieve high-accuracy multi-class classification [15]; Maçin et al. designed a lightweight KidneyNeXt network that balances classification accuracy with clinical deployment feasibility [16]. In addition, Özbay et al. employed self-supervised learning methods to enhance the robustness of kidney tumor classification in small-sample scenarios [17], while Bingol et al. integrated feature selection with deep networks to realize automatic classification of kidney CT images [18]. At the same time, Zhang et al. and Rana et al. provided comprehensive reviews that systematically summarize the applications and challenges of deep learning in kidney disease imaging and broader medical image classification [14,19]. These studies have laid an important foundation for the multi-window enhancement and texture prior fusion approach proposed in this paper.

### 2.2 Contrast enhancement and multi-window fusion strategies in imaging analysis

In computed tomography (CT) analysis, the configuration of window width (WW) and window level (WL) plays a critical role in lesion visualization and model classification performance. Traditional fixed window settings often fail to balance both soft tissues and high-density structures, thereby limiting the discriminative capacity of deep models. To address this issue, researchers have proposed a series of automated and optimized approaches, such as interpretable automatic window parameter learning for personalized adjustment [20], or trainable window optimization modules to enhance feature sensitivity [21,22]. In addition, multi-window fusion and window-mixing algorithms have been widely applied. For example, the RADIO algorithm integrates multiple window settings to improve clinical diagnostic efficiency [23], while organ-specific windowing in whole-body trauma CT has been shown to accelerate reading speed and increase diagnostic accuracy [24]. These studies demonstrate that contrast enhancement through window optimization not only improves visualization but also provides richer and more discriminative input features for deep learning models.

In recent years, the advantages of multi-window techniques have been further validated in specific lesion detection and segmentation tasks. For instance, in intracranial hemorrhage studies, the combination of bone windows and subdural windows is commonly employed to improve hemorrhage region detection accuracy [25,26]. Meanwhile, HU-to-RGB conversion and automatic window selection strategies have significantly enhanced classification performance in non-contrast CT [27]. On low-energy monochromatic images, optimized window configurations have been leveraged to achieve contrast enhancement for inflammation-related imaging [28]; in pulmonary nodule tasks, multi-window inputs combined with uncertainty-guided mechanisms have demonstrated superior segmentation of heterogeneous lesion types [29]. In summary, research on multi-window fusion and contrast enhancement continues to drive CT imaging from traditional manual dependence toward intelligent and automated approaches, providing critical support for improving the robustness and generalization of subsequent deep learning models.

Beyond the medical imaging domain, several recent studies have explored related enhancement and feature-decoupling strategies in remote sensing and biomedical segmentation tasks, offering methodological guidance for multi-domain feature representation. Li et al. proposed Euclidean affinity-augmented and frequency-decoupled networks for remote sensing image segmentation [30–32], which demonstrated the effectiveness of combining spatial-frequency cues for improved discriminability. Similarly, Tong et al. introduced dynamic frequency-decoupled refinement and edge-enhanced architectures for medical image segmentation [33,34]. Although these works focus on remote sensing and polyp segmentation rather than CT imaging, their methodological innovations—particularly in dual-domain fusion and frequency-based enhancement—provide valuable insights that align with the motivation of our adaptive dual-window and texture-fusion design.

### 2.3 Texture priors and structural feature modeling in medical imaging

Texture features and structural priors are widely regarded as key factors for enhancing the discriminative power and interpretability of models in medical image analysis. Early studies exploited texture priors learned from full-dose CT data for Bayesian reconstruction of low-dose CT, effectively improving image quality and preserving tissue details [35]. Further clinical investigations have demonstrated that spatial texture analysis can sensitively capture intra-tumor heterogeneity, thereby providing radiologists with additional quantitative information [36]. In cardiac imaging, Xu et al. proposed a method that integrates radiomic texture features with deep learning for automated detection of myocardial infarction, significantly improving the diagnostic efficiency and accuracy of MRI [37]. At the same time, the incorporation of edge and structural priors into medical image restoration has facilitated better capture of morphological features and the recovery of missing details [38].

In recent years, an increasing number of studies have focused on translating texture analysis methods into clinically applicable tools. Corrias et al. summarized the key aspects of texture imaging in radiological practice, emphasizing its importance for clinicians [39]. In magnetic resonance imaging, Materka provided a systematic review of texture analysis methods, covering statistical, structural, and filtering approaches [40]. The development of radiomics has further advanced the use of texture features in multimodal imaging analysis, with Van Timmeren et al. presenting a comprehensive research workflow and critical reflections [41]. At the same time, deep learning research has shown that reducing a model’s reliance on and bias toward texture can enhance the robustness of semantic segmentation tasks [42]. Fekri-Ershad conducted a comprehensive review of image texture analysis methods, offering researchers a systematic theoretical framework and guidance for methodological choices [43]. In summary, the modeling of texture and structural features not only provides a solid foundation for improving the performance of automated analysis but also establishes theoretical support for the design of deep learning models that incorporate prior knowledge.

## 3 Method

### 3.1 Ethics Statement

This article does not require ethical approval as the data used are open source data.

### 3.2 Overall model architecture

**Fig 1:**
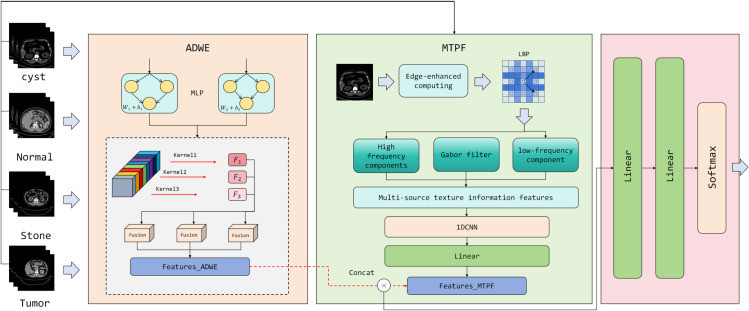
Overall framework of the proposed kidney CT classification model. The ADWE module adaptively enhances contrast through dual-window fusion, while the MTPF module integrates edge, LBP, and Gabor priors for multi-scale texture representation, and the fused features are fed into the classifier for final prediction.

The overall framework of the proposed model is designed to enhance the accuracy and robustness of kidney CT image classification, with its core innovation lying in the integration of adaptive dual-window enhancement and multi-scale texture prior fusion as two complementary strategies. Specifically, the input CT image is first processed by the adaptive dual-window enhancement module to generate two complementary views: one emphasizes low-contrast regions such as soft tissues and cysts, while the other highlights high-density structures such as kidney stones. This provides the network with richer and more diverse discriminative information at the input stage. Subsequently, the model incorporates multi-scale texture priors, where texture feature maps generated through edge detection, local binary patterns, and filter responses are fused with the original image features to capture morphological variations and boundary details across different scales. Ultimately, the contrast-enhanced information from the dual-window module and the fine-grained structural information introduced by texture priors act synergistically in the classification backbone, enabling the model to achieve stronger discriminative capability and higher robustness when identifying cysts, stones, tumors, and normal tissues. The overall design is lightweight and deployment-friendly, while significantly improving the classification performance of multi-class kidney CT images. Its overall architecture is shown in Fig 1.

### 3.3 Adaptive dual-window enhancement

The proposed Adaptive Dual-Window Enhancement module is designed to fully exploit the representation of kidney CT images across different gray-level ranges in order to improve the overall discriminative capability of classification tasks. Conventional CT images are typically displayed with fixed window width (WW) and window level (WL) settings. However, such a single configuration often fails to simultaneously preserve contrastive features of both soft tissue lesions and high-density abnormalities, which inevitably limits classification performance. To address this issue, we design a learnable dual-window mechanism that generates two complementary enhanced views in parallel, enabling the network to simultaneously focus on low-contrast regions (e.g., cysts and tumors in soft tissues) and high-contrast regions (e.g., kidney stones in hard tissues). This strategy allows the model to construct more comprehensive and robust discriminative representations during feature extraction.

Formally, given an input image $X\in {\mathbb{R}}^{H\times W}$, we define the window function as a differentiable nonlinear transformation:

$$f(x;WL,WW)=\frac{1}{2}\left[\tanh\left(\alpha \cdot \frac{x-WL}{WW}\right)+1\right],$$ (1)

where *x* denotes the pixel intensity, *WL* and *WW* represent the window level and window width parameters, respectively, and *α* is a learnable scaling factor controlling the smoothness of the transformation. To obtain two distinct views, we apply two sets of adaptive parameters $\left(WL_{1},WW_{1}\right)$ and $\left(WL_{2},WW_{2}\right)$ to the same input, thereby producing the parenchymal-window image *X*_1_ and the high-density-window image *X*_2_:

$$X_{1}=f(X;WL_{1},WW_{1}),\;X_{2}=f(X;WL_{2},WW_{2}).$$ (2)

**Fig 2:**
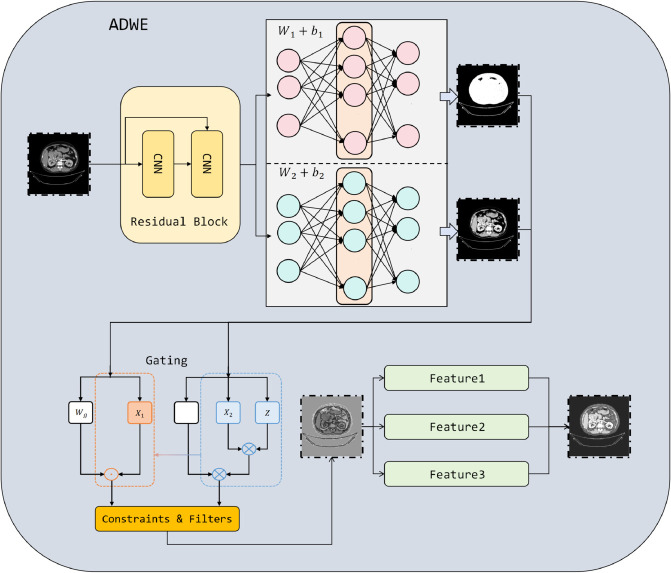
Illustration of the proposed adaptive dual-window enhancement module. The input CT image is processed through residual learning, dual-window adaptive mapping, and gated fusion with constraints and multi-scale filters, generating complementary representations for robust kidney CT classification.

To ensure that the learned window settings remain physically reasonable during training, we introduce bounded constraints formalized as a projection operation:

$$WL_{i}\leftarrow \text{clip}(WL_{i},a,b),\;WW_{i}\leftarrow \text{clip}(WW_{i},c,d),\;i=1,2,$$ (3)

where [*a*,*b*] and [*c*,*d*] denote the valid ranges for window level and window width, respectively. The two enhanced views *X*_1_ and *X*_2_ maintain spatial consistency but exhibit complementary gray-level contrasts, thereby providing the classifier with differentiated features. The detailed architecture of this module is shown in Fig 2.

For information fusion, we design a lightweight gating mechanism:

$$Z=\sigma (W_{g}\cdot [X_{1},X_{2}]+b_{g}),$$ (4)

where $\left[X_{1},X_{2}\right]$ denotes channel concatenation, *W*_*g*_ and *b*_*g*_ are learnable parameters, and σ(·) is the Sigmoid activation. The fusion weight *Z* is applied to the enhanced features to obtain the final representation:

$$X^{*}=Z⊙X_{1}+(1-Z)⊙X_{2},$$ (5)

where ⊙ denotes element-wise multiplication. In this manner, the model adaptively selects the most discriminative view combination according to the input characteristics, thereby avoiding information loss inherent in single fixed window settings.

To further strengthen discriminative ability, we incorporate multi-scale convolution operations in the feature domain:

$$F=\sum_{k\in \{3,5,7\}}W_{k}*X^{*},$$ (6)

where * denotes convolution and *W*_*k*_ corresponds to kernels with different receptive fields. This design simultaneously captures local details and global structural patterns, better adapting to the complex morphologies of renal lesions.

Additionally, to ensure that the dual-window enhancement statistically covers the major gray-level distributions, we impose a weighted histogram matching constraint:

$$ℋ(X^{*})=\lambda_{1}\cdot ℋ(X_{1})+\lambda_{2}\cdot ℋ(X_{2}),\;\lambda_{1}+\lambda_{2}=1,$$ (7)

where ℋ(·) denotes the histogram distribution and $\lambda_{1},\lambda_{2}$ are fusion coefficients. This constraint enables the fused representation to preserve statistical characteristics of both low- and high-density regions, thereby improving overall discriminability.

Finally, the enhanced features *F* are fed into the classification backbone for deep feature extraction and decision-making. Through adaptive contrast adjustment, multi-view information fusion, and multi-scale feature modeling, the ADWE module significantly strengthens the model’s ability to distinguish cysts, stones, tumors, and normal tissues in 2D CT images, demonstrating superior robustness and generalization capability.

### 3.4 Multi-scale texture prior fusion

**Fig 3:**
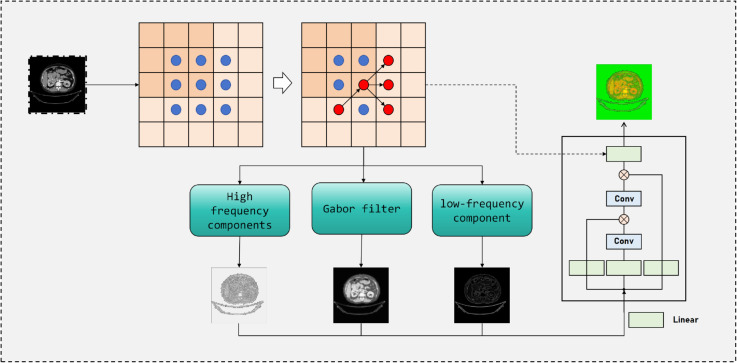
Illustration of the multi-scale texture prior fusion module. The input CT image is decomposed into edge, frequency, and Gabor-based texture components, which are fused through convolutional layers to generate robust multi-scale representations for kidney CT classification.

To further enhance the fine-grained discriminative capability of kidney CT classification, we propose a Multi-scale Texture Prior Fusion module on top of the Adaptive Dual-Window Enhancement. While ADWE emphasizes contrastive differences between soft tissues and high-density structures from the perspective of gray-level adjustment, MTPF introduces complementary discriminative cues from the texture and structural domain, enabling the model to better differentiate subtle variations among cysts, stones, tumors, and normal tissues. Specifically, we first extract multi-scale texture prior maps from the enhanced image X^*^ and then integrate them with backbone representations through a feature fusion mechanism, thereby achieving complementary enhancement between global contrast and local texture details. The module architecture is shown in Fig 3.

Given the enhanced image $X^{*}\in {\mathbb{R}}^{H\times W}$, we first compute its edge-enhanced map *E* using the Laplacian operator:

$$E=X^{*}*K_{lap},\;K_{lap}=[\begin{array}{ccc} 0 & -1 & 0\\ -1 & 4 & -1\\ 0 & -1 & 0 \end{array}],$$ (8)

where * denotes convolution and *K*_*lap*_ is the Laplacian kernel. This edge map highlights lesion boundaries, thereby assisting in distinguishing morphologically similar abnormalities with distinct edge characteristics.

Next, we incorporate Local Binary Pattern as a texture prior. For any pixel *p*, the LBP encoding is defined as:

$$LBP(p)=\sum_{k=0}^{P-1}s(I_{k}-I_{p})\cdot 2^{k},\;s(z)=\left\{ \begin{array}{cc} 1, & z\geq 0\\ 0, & z<0 \end{array}\right.,$$ (9)

where *I*_*p*_ is the gray value of the central pixel, *I*_*k*_ is the intensity of its neighborhood samples, and *P* denotes the number of sampled points. LBP effectively captures differences between homogeneous textures of cysts and irregular complex textures of tumors.

In addition, we employ multi-scale Gabor filters to extract frequency-domain features, defined as:

$$G_{u,v}(x,y)=\exp\left(-\frac{x^{\prime 2}+\gamma^{2}y^{\prime 2}}{2\sigma^{2}}\right)\cos\left(2\pi \frac{x^{\prime }}{\lambda }+\phi \right),$$ (10)

where $x^{\prime }=x\cos\theta \;+\;y\sin\theta$, $y^{\prime }=-x\sin\theta \;+\;y\cos\theta$, and the parameters λ,θ,ϕ,σ,γ correspond to wavelength, orientation, phase, scale, and aspect ratio, respectively. The multi-scale and multi-orientation Gabor responses yield a set of multi-channel feature maps $\left\{G_{u,v}\right\}$, which capture high-frequency details of stones and low-frequency structures of soft tissues.

For unified modeling, we concatenate *E*, *LBP*, and $\left\{G_{u,v}\right\}$ into a texture prior tensor *T*:

$$T=\text{Concat}(E,LBP,G_{u,v}),\;T\in {\mathbb{R}}^{H\times W\times C_{t}},$$ (11)

where *C*_*t*_ denotes the number of texture channels. The tensor *T* is then fed into a lightweight convolutional module for dimensionality reduction and feature compression:

$$T^{\prime }=\delta (W_{t}*T+b_{t}),$$ (12)

where *W*_*t*_ and *b*_*t*_ are learnable parameters, and δ(·) is a nonlinear activation function. This step suppresses noise while highlighting stable texture patterns.

During feature fusion, we adopt a channel attention mechanism to guide complementary integration of texture and backbone features. Let $F\in {\mathbb{R}}^{H^{\prime }\times W^{\prime }\times C_{f}}$ denote the backbone features and $T^{\prime }$ the texture features, the fusion is defined as:

$$\alpha =\sigma \left(W_{\alpha }\cdot \text{GAP}(T^{\prime })\right),\;\beta =\sigma \left(W_{\beta }\cdot \text{GAP}(F)\right),$$ (13)

$$F^{*}=\alpha ⊙F+\beta ⊙T^{\prime },$$ (14)

where GAP(·) denotes global average pooling, σ(·) is the Sigmoid function, and ⊙ indicates channel-wise multiplication. This design allows the network to adaptively select the most discriminative cues from either texture priors or backbone representations.

To further enhance multi-scale representation, we apply pyramid pooling on the fused features:

$$F_{ms}=\sum_{s\in \{1,2,4\}}U_{s}\left(P_{s}(F^{*})\right),$$ (15)

where $P_{s}(\cdot )$ denotes pooling at scale *s* and $U_{s}(\cdot )$ represents upsampling to the original resolution. This ensures the simultaneous capture of local lesion details and global structural context.

Moreover, to ensure statistical consistency between texture priors and the original input, we impose a distribution matching constraint using Maximum Mean Discrepancy (MMD):

$${\text{MMD}}^{2}(T^{\prime },X^{*})={\left\|\frac{1}{n}\sum_{i=1}^{n}\phi (T_{i}^{\prime })-\frac{1}{m}\sum_{j=1}^{m}\phi (X_{j}^{*})\right\|}^{2},$$ (16)

where ϕ(·) is a kernel mapping function, and *n* and *m* denote the sample sizes. This constraint enforces distributional alignment between texture features and the original image, thus mitigating overfitting risks.

Finally, the fused multi-scale representation *F*_*ms*_ is fed into the classification backbone to form the joint representation:

$$H=\Psi (F_{ms}),$$ (17)

where Ψ(·) denotes the backbone classifier. Through the MTPF module, the model incorporates rich texture and structural priors on top of gray-level contrast enhancement, resulting in more comprehensive and robust classification representations. In particular, the module demonstrates superior discriminative capability for subtle differences, and when combined with ADWE, the overall framework achieves multi-dimensional feature modeling for kidney CT images, thereby substantially improving classification performance.

## 4 Dataset and evaluation metrics

### 4.1 Dataset

**Fig 4:**
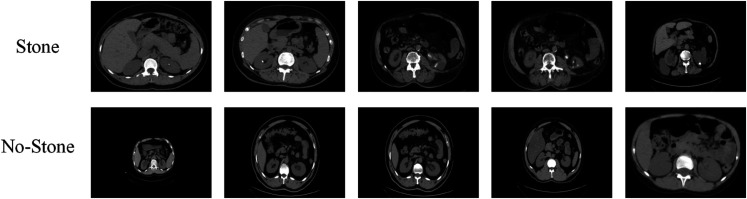
Dataset 1 example display.

This study utilized two publicly available kidney CT imaging datasets to support both multi-class and binary classification tasks. The first dataset was provided by medical centers in Sulaymaniyah and Ranya, Iraq, with a specific focus on kidney stone detection. It consists of two categories: “stone present” and “stone absent.” The dataset originally contained 3,364 raw CT images, which underwent a comprehensive data augmentation process including random rotation ($\pm 15^{\circ }$), horizontal and vertical flipping, random translation, scaling (0.9–1.1), brightness and contrast jittering, and gamma correction. These operations were applied probabilistically to diversify imaging conditions and anatomical orientations, thereby mitigating overfitting and improving generalization. The final augmented dataset contained a total of 38,821 images, significantly enhancing its suitability and robustness for model training. All images were annotated and validated by experienced medical experts, ensuring the accuracy and clinical reliability of the labels. Beyond capturing diverse real-case presentations, the augmented dataset also expanded the training space, making it an essential resource for the development and validation of deep learning algorithms, while simultaneously offering considerable potential for medical education and research. This dataset therefore establishes a solid basis for advancing computer-aided diagnosis of kidney stones. An example of the data is shown in Fig 4.

**Fig 5:**
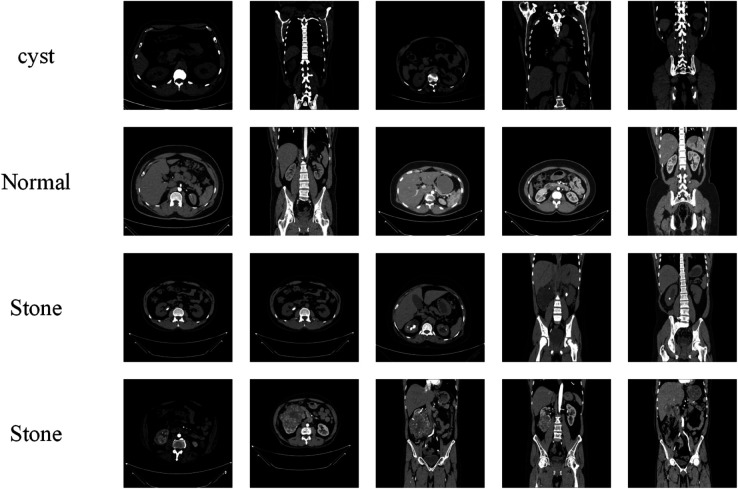
Dataset 2 example display.

The second dataset was collected from the PACS systems of several hospitals in Dhaka, Bangladesh, and comprises CT scans of patients with confirmed diagnoses of normal kidneys, cysts, stones, and tumors. Both coronal and axial slices were included, covering contrast-enhanced and non-contrast studies to ensure diversity in imaging modalities and diagnostic features. After removing patient information and metadata, the DICOM images were converted into lossless JPG format. Each image was then verified by radiologists and medical technologists to guarantee data integrity. Ultimately, a rigorously quality-controlled multi-class CT dataset was established, consisting of 12,446 images, including 5,077 normal, 3,709 cyst, 1,377 stone, and 2,283 tumor cases, thereby providing a reliable foundation for automated kidney disease classification. Similarly, an example display result of this dataset is given, as shown in Fig 5.

### 4.2 Evaluation metric

To comprehensively evaluate the performance of the proposed model in kidney CT classification tasks, five commonly used metrics were selected, namely classification accuracy (Accuracy, ACC), precision (Precision), recall (Recall), F1-score (F1), and the area under the receiver operating characteristic curve (Area Under Curve, AUC). These metrics reflect the discriminative capability and robustness of the model from different perspectives in both multi-class and binary classification tasks, thereby ensuring the reliability and clinical significance of the results.

First, accuracy (ACC) measures the overall proportion of correctly predicted samples and serves as the most intuitive evaluation metric. It is calculated as the ratio of correctly classified samples to the total number of samples, thus reflecting the model’s overall performance. However, for imbalanced datasets, relying solely on accuracy may mask poor predictions on minority classes. The formula is given as:

$$ACC=\frac{TP+TN}{TP+TN+FP+FN},$$ (18)

where *TP*, *TN*, *FP*, and *FN* denote true positives, true negatives, false positives, and false negatives, respectively.

Second, precision evaluates the proportion of truly positive samples among all samples predicted as positive. In kidney stone or tumor detection tasks, a high precision indicates a low false positive rate, which is particularly important for reducing unnecessary clinical interventions. It is defined as:

$$Precision=\frac{TP}{TP+FP}.$$ (19)

Third, recall measures the proportion of correctly identified positive samples among all actual positive cases. In medical image analysis, achieving high recall is especially critical, as it directly relates to the completeness of lesion detection, and missed diagnoses may lead to severe consequences for patient care. The formula is:

$$Recall=\frac{TP}{TP+FN}.$$ (20)

Since there often exists a trade-off between precision and recall, the F1-score is introduced as their harmonic mean, providing a balanced evaluation of the model’s performance in terms of both false alarms and missed detections. The formula is:

$$F1=2\times \frac{Precision\times Recall}{Precision+Recall}.$$ (21)

A higher F1-score indicates that the model can achieve effective detection while simultaneously reducing false positives, which is particularly suitable for medical datasets with imbalanced class distributions.

Finally, the area under the receiver operating characteristic curve (AUC) is an important indicator of the overall discriminative power of the model across different threshold settings. A higher AUC value, closer to 1, indicates better performance in distinguishing between positive and negative cases. AUC is defined as the integral of the true positive rate (TPR) with respect to the false positive rate (FPR):

$$AUC=\int_{0}^{1}TPR(FPR)\;d(FPR),$$ (22)

where

$$TPR=\frac{TP}{TP+FN},\;FPR=\frac{FP}{FP+TN}.$$ (23)

In medical classification tasks, AUC comprehensively reflects the robustness of the model under varying decision thresholds and has therefore become a key standard for evaluating classification performance.

## 5 Experimental results and analysis

### 5.1 Experimental setup

To validate the effectiveness of the proposed method in kidney CT classification tasks, we conducted a systematic evaluation under a unified experimental environment. During training, all images were divided into training, validation, and testing sets with a ratio of 7:1.5:1.5, ensuring that the model had sufficient training samples while also achieving reliable generalization performance on independent validation and test sets. All experiments were performed under the same hardware environment to ensure result comparability. Training was performed for a total of 100 epochs, and multiple evaluation metrics were monitored throughout the process to assess the classification capability of the model.

For the implementation, ConvNeXtV2 was adopted as the backbone network, integrated with the proposed Adaptive Dual-Window Enhancement and Multi-Scale Texture Prior Fusion modules for feature extraction and classification. Specifically, the ConvNeXtV2-Tiny variant was utilized, which contains four stages with depths of [3, 3, 9, 3] and an embedding dimension of 96. Standard data augmentation strategies, including random rotation ($\pm 15^{\circ }$), horizontal and vertical flipping, random cropping, and brightness/contrast jittering were employed to enhance generalization. All images were resized to 224×224 and normalized using z-score normalization with dataset-wide mean and standard deviation. The AdamW optimizer was employed, combined with a cosine annealing learning rate scheduler to improve training stability. The batch size was set to 32, the initial learning rate was $1\;\times \;10^{-4}$, and the weight decay coefficient was set to $1\;\times \;10^{-5}$. All experiments were carried out on a workstation equipped with an NVIDIA RTX 4090 GPU under Ubuntu 22.04, using PyTorch 2.0 as the deep learning framework. The detailed experimental configurations are summarized in Table 1.

**Table 1:** Experimental setup and training hyperparameter configurations.

Item	Configuration
Algorithm framework	ConvNeXtV2 + ADWE + MTPF
Backbone network	ConvNeXtV2-Tiny (depths [3,3,9,3], dim 96)
Training epochs	100
Data split ratio	7 : 1.5 : 1.5
Optimizer	AdamW
Initial learning rate	$1\times 10^{-4}$
Batch size	32
Weight decay	$1\times 10^{-5}$
Learning rate scheduler	Cosine annealing
Data augmentation	Rotation, flip, crop, brightness/contrast jitter
Normalization	z-score (global mean/std)
Input size	224×224
Hardware environment	NVIDIA RTX 4090 GPU, 24GB VRAM
Operating system	Ubuntu 22.04
Deep learning framework	PyTorch 2.0

### 5.2 Comparative experimental results

To comprehensively validate the effectiveness of the proposed method, we conducted comparative experiments against a variety of mainstream image classification algorithms, including ResNet50, VGG19, ConvNeXt, ResNeXt, Vision Transformer, and Swin Transformer. In addition, representative approaches specifically designed for medical image analysis, such as MedMamba, DM-CNN, HiFuse, and Res-MGCA-SE, were also included to ensure the comparisons are well-targeted in clinical scenarios. Through these comparative studies, we are able to more clearly assess the advantages and performance improvements of the proposed model over different categories of methods. The experimental results are summarized in Table 2.

**Table 2:** Performance comparison of different methods on the kidney CT two classification task (best results are highlighted in bold).

Method	ACC	Precision	Recall	F1-Score	AUC
VGG19 [44]	0.9401	0.9312	0.9280	0.9296	0.9801
ResNet50 [45]	0.9554	0.9531	0.9465	0.9498	0.9885
ResNeXt [46]	0.9583	0.9568	0.9512	0.9540	0.9893
ConvNeXt [47]	0.9632	0.9621	0.9550	0.9585	0.9911
Vision Transformer [48]	0.9665	0.9655	0.9583	0.9619	0.9924
Swin Transformer [49]	0.9712	0.9718	0.9627	0.9672	0.9951
HiFuse [50]	0.9690	0.9689	0.9604	0.9646	0.9936
DM-CNN [51]	0.9605	0.9589	0.9487	0.9538	0.9907
Res-MGCA-SE [52]	0.9741	0.9743	0.9641	0.9692	0.9968
MedMamba [53]	0.9756	0.9762	0.9650	0.9706	0.9977
**Ours**	**0.9802**	**0.9913**	**0.9662**	**0.9786**	**0.9989**

In the binary classification task, both mainstream deep learning models and medical image–oriented methods demonstrated strong classification capabilities; however, their overall performance was still constrained by insufficient input contrast and the lack of fine-grained texture representation. As shown in Table 2, classical convolutional neural networks such as VGG19 and ResNet50 exhibited relatively stable performance in terms of overall accuracy and AUC, but encountered misclassification issues when distinguishing kidney stones from normal tissues. With the evolution of network architectures, ConvNeXt, ResNeXt, and Transformer-based models achieved significant improvements through enhanced feature modeling capabilities. In particular, the Swin Transformer and medical-specific models such as MedMamba and Res-MGCA-SE attained near-optimal performance in accuracy and F1-score, indicating that incorporating hierarchical modeling and domain priors has a positive impact on medical CT classification tasks.

In comparison, the proposed method achieved the best results across all evaluation metrics, with an accuracy of 0.9802 and an AUC of 0.9989. This superior performance can be attributed to the synergistic effects of the Adaptive Dual-Window Enhancement and Multi-Scale Texture Prior Fusion modules. Specifically, ADWE dynamically adjusts multiple contrast-enhanced views, effectively mitigating the information loss problem associated with traditional single-window settings, while MTPF introduces edge, texture, and frequency-domain priors, enabling the model to establish more robust decision boundaries between soft and hard tissue characteristics. The combination of these two modules allows the model to better capture global density differences and local texture patterns, thereby delivering superior discriminative performance in kidney CT binary classification compared with existing approaches. Furthermore, the experimental results of the four categories are given, as shown in Table 3.

**Table 3:** Performance comparison of different methods on the kidney CT four-class classification task (best results are highlighted in bold).

Method	ACC	Precision	Recall	F1-Score	AUC
VGG19	0.8123	0.8245	0.7321	0.7754	0.9412
ResNet50	0.8356	0.8478	0.7559	0.7994	0.9536
ResNeXt	0.8467	0.8582	0.7688	0.8110	0.9614
ConvNeXt	0.8521	0.8665	0.7742	0.8176	0.9647
Vision Transformer	0.8610	0.8733	0.7834	0.8260	0.9691
Swin Transformer	0.8694	0.8805	0.7917	0.8344	0.9723
HiFuse	0.8587	0.8714	0.7802	0.8233	0.9682
DM-CNN	0.8475	0.8596	0.7693	0.8121	0.9621
Res-MGCA-SE	0.8739	0.8862	0.7965	0.8392	0.9745
MedMamba	0.8770	0.8891	0.8003	0.8430	0.9759
**Ours**	**0.8821**	**0.8956**	**0.8055**	**0.8438**	**0.9801**

As shown in Table 3, in the kidney CT four-class classification task, traditional convolutional networks such as VGG19 and ResNet50 exhibit certain limitations in terms of accuracy and recall. With the evolution of network architectures, methods such as ConvNeXt, Vision Transformer, and Swin Transformer achieve improved overall performance, while medical image–oriented models such as Res-MGCA-SE and MedMamba further demonstrate domain-specific advantages. In contrast, the proposed method achieves the best results across all five evaluation metrics, with an accuracy of 0.8821 and an AUC of 0.9801. These results indicate that the combination of Adaptive Dual-Window Enhancement and Multi-Scale Texture Prior Fusion not only compensates for the shortcomings of single-feature representations but also enables more robust discriminative capability in multi-class lesion differentiation, thereby effectively enhancing the classification performance of kidney CT images.

### 5.3 Ablation experiment results

To further validate the contribution of each module within the overall framework, we designed a systematic ablation study by progressively removing and combining the Adaptive Dual-Window Enhancement and Multi-Scale Texture Prior Fusion modules. In this manner, the individual and joint impacts of these components on feature representation capability and classification performance can be more clearly revealed. This paper presents the experimental results of two datasets, as shown in Table 4.

**Table 4:** Comparison of ablation study results (baseline is ConvNeXtV2; best results are highlighted in bold).

Method	ACC	Precision	Recall	F1-Score	AUC
**Binary Classification Dataset**					
Baseline (ConvNeXtV2)	0.9704	0.9787	0.9578	0.9680	0.9943
+ADWE	0.9741	0.9832	0.9605	0.9716	0.9961
+MTPF	0.9775	0.9876	0.9638	0.9756	0.9975
**Ours**	**0.9802**	**0.9913**	**0.9662**	**0.9786**	**0.9989**
**Four-Class Classification Dataset**					
Baseline (ConvNeXtV2)	0.8539	0.8624	0.7660	0.7946	0.9758
+ADWE	0.8647	0.8741	0.7782	0.8235	0.9773
+MTPF	0.8730	0.8845	0.7920	0.8361	0.9787
**Ours**	**0.8821**	**0.8956**	**0.8055**	**0.8438**	**0.9801**

**Fig 6:**
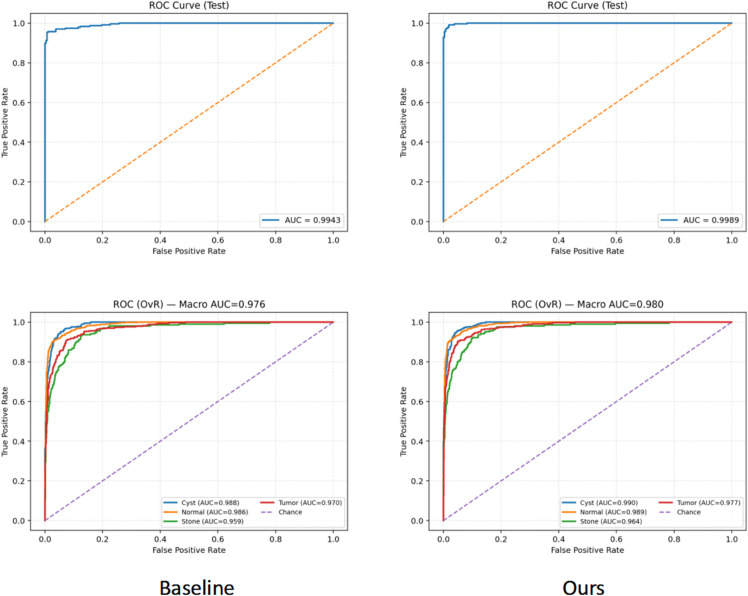
Comparison of ROC curves between the baseline and the algorithm proposed in this paper.

As shown in Table 4, the progressive introduction of Adaptive Dual-Window Enhancement and Multi-Scale Texture Prior Fusion consistently improves performance in both binary and four-class classification tasks, indicating that the two proposed modules effectively complement the feature representation capability of the baseline network. Specifically, ADWE enhances the model’s perception of different grayscale contrast regions by introducing complementary windows at the input stage, while MTPF further characterizes fine-grained lesion differences through multi-scale texture priors. Their synergistic integration enables the final model to achieve superior results across multiple dimensions, including accuracy, precision, recall, F1-score, and AUC, thereby validating the rationality and effectiveness of the proposed design. In order to intuitively express the effectiveness of this algorithm, this paper also gives the ROC curves of the baseline and this algorithm, as shown in Fig 6.

As illustrated by the ROC curves, the proposed method demonstrates superior discriminative capability compared with the baseline model in both binary and four-class classification tasks. The overall curves are closer to the upper-left corner, indicating that the model maintains high stability in distinguishing positive and negative samples across different thresholds. The combination of Adaptive Dual-Window Enhancement and Multi-Scale Texture Prior Fusion enables complementary strengths in global contrast modeling and local fine-grained feature representation, thereby enhancing the generalization ability and robustness of the model in complex kidney CT classification scenarios.

### 5.4 t-SNE experimental results analysis

**Fig 7:**
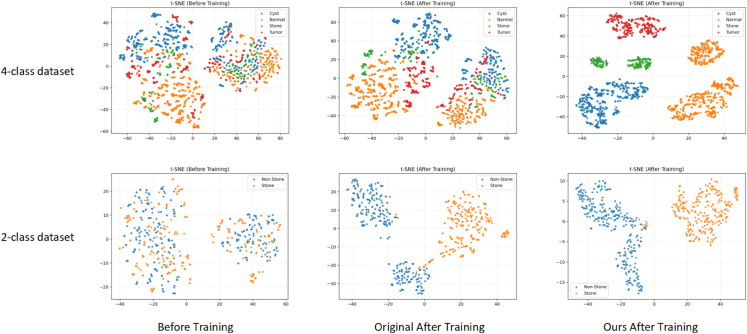
t-SNE analysis experimental results, including the t-SNE experimental results before training, the experimental results after training of the original baseline training, and the experimental results of the algorithm in this paper.

In this section, we provide a t-SNE comparison chart before and after training, which intuitively demonstrates the classification effectiveness of the algorithm proposed in this paper. The experimental results are shown in Fig 7.

As shown in the t-SNE visualization results in Fig 7, the samples exhibit significant intermixing before training, with feature distributions of different categories displaying substantial overlap, making it difficult to establish effective inter-class boundaries. After training with the baseline model, the feature distributions become gradually more separable; however, partial overlaps still remain among certain categories. This is particularly evident in cases such as kidney cysts and tumors, where tissue characteristics are highly similar, limiting the model’s ability to discriminate between complex classes.

In contrast, the proposed method demonstrates clearer feature clustering patterns in both binary and four-class classification tasks. Different categories form compact intra-class clusters and well-separated inter-class boundaries in the low-dimensional feature space. This indicates that the Adaptive Dual-Window Enhancement effectively improves the model’s sensitivity to grayscale variations, while the Multi-Scale Texture Prior Fusion further strengthens the representation of fine-grained structural features. The synergistic effect of these modules enables the model to achieve stronger discriminative power in the latent feature space, thereby providing more robust and distinguishable feature representations for subsequent classification tasks.

To further provide quantitative evidence of the clustering quality observed in the t-SNE visualizations, three widely used cluster evaluation metrics were calculated on the extracted feature embeddings: the Silhouette Score (higher is better), the Calinski–Harabasz Index (higher indicates better separation), and the Davies–Bouldin Index (lower indicates better compactness), as shown in Table 5.

**Table 5:** Quantitative cluster evaluation metrics on t-SNE feature embeddings for different models. Higher Silhouette and Calinski–Harabasz scores indicate better class separation, while lower Davies–Bouldin values denote tighter intra-class clustering.

Method	Silhouette	Calinski–Harabasz	Davies–Bouldin
Before Training	0.21	185.7	1.98
Baseline Model	0.42	356.3	1.25
**Ours (ADWE + MTPF)**	**0.67**	**624.8**	**0.78**

As shown in Table 5, the proposed method achieves the highest Silhouette and Calinski–Harabasz scores while maintaining the lowest Davies–Bouldin index across both datasets, confirming that the learned feature space exhibits superior intra-class compactness and inter-class separability.

### 5.5 Grad-Cam and confusion matrix analysis

**Fig 8:**
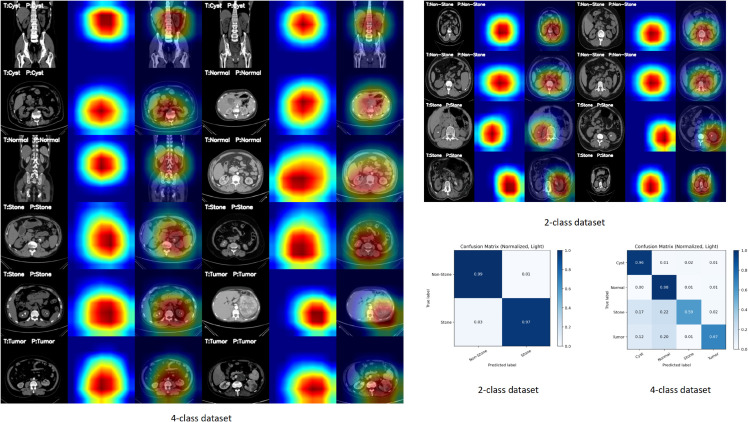
Grad-cam visualization and confusion matrix analysis.

In the last part of the experiment, the Grad-CAM visualization and confusion matrix analysis of the proposed algorithm on two datasets are given, and the experimental results are shown in Fig 8.

As shown in the Grad-CAM visualization results in Fig 8, the proposed method is able to more accurately focus on lesion-related key regions rather than being distracted by background or irrelevant tissues. In the binary classification task, the heatmaps primarily concentrate on the discriminative areas corresponding to kidney stones or normal tissues. In the four-class classification task, the model successfully highlights the lesion regions associated with different pathological types, including cysts, tumors, and stones. This demonstrates that the introduction of Adaptive Dual-Window Enhancement and Multi-Scale Texture Prior Fusion endows the model with stronger regional perception capability, thereby improving both the interpretability and reliability of the classification results.

Meanwhile, the confusion matrix results show that the model maintains high recognition accuracy across most categories, with misclassifications primarily occurring between clinically similar classes, such as cysts and tumors. Overall, the proposed method exhibits more robust discriminative ability in both binary and four-class classification tasks, significantly reducing inter-class confusion. These findings not only validate the advantages of the model in feature learning but also highlight its high clinical applicability and potential utility in real-world medical imaging scenarios.

## 6 Restriction analysis

Because CT images are often subject to noise, this paper also conducted a restrictive analysis of the proposed model and the baseline. We compared the experimental results by adding different noise intensities to the four-category classification. The experimental results are shown in Table 6.

**Table 6:** Comparison of robustness experiment results under different noise intensities (decline indicates the degree of performance drop; best results are highlighted in bold).

Method	ACC	Precision	Recall	F1-Score	AUC	Decline
Baseline (σ=0)	0.8539	0.8624	0.7660	0.7946	0.9758	0
Ours (σ=0)	**0.8821**	**0.8956**	**0.8055**	**0.8438**	**0.9801**	0
Baseline (σ=0.01)	0.8475	0.8551	0.7594	0.7893	0.9712	–0.0046
Ours (σ=0.01)	**0.8764**	**0.8890**	**0.7991**	**0.8385**	**0.9768**	–0.0033
Baseline (σ=0.05)	0.8327	0.8412	0.7426	0.7881	0.9627	–0.0131
Ours (σ=0.05)	**0.8653**	**0.8776**	**0.7872**	**0.8299**	**0.9705**	–0.0096
Baseline (σ=0.1)	0.8159	0.8265	0.7241	0.7719	0.9516	–0.0242
Ours (σ=0.1)	**0.8510**	**0.8642**	**0.7750**	**0.8172**	**0.9634**	–0.0167

As shown in Table 6, with the gradual increase of noise intensity, both the baseline model and the proposed method exhibit a certain degree of performance degradation, indicating that noise in CT images indeed interferes with feature extraction and classification. However, compared with the baseline, the proposed method consistently maintains higher accuracy, precision, recall, and AUC across all noise levels, while experiencing significantly smaller performance declines. This demonstrates that the introduction of Adaptive Dual-Window Enhancement and Multi-Scale Texture Prior Fusion effectively improves the model’s adaptability to input perturbations, thereby preserving stable discriminative capability under low signal-to-noise ratio conditions.

Further analysis reveals that under higher noise levels (σ=0.05 and σ=0.1), the proposed method still exhibits stronger robustness than the baseline model. This result indicates that the proposed modules not only enhance the feature representation ability of the network but also strengthen its capability to suppress redundant information and noise interference. In practical medical applications, this implies that even in the presence of scanning noise or suboptimal image quality, the model can maintain high clinical reliability, thus providing a more robust guarantee for intelligent auxiliary diagnosis of kidney diseases.

Furthermore, this paper gives a training cost analysis, as shown in Table 7.

**Table 7:** Comparison of computational cost and training efficiency among different models under identical hardware (NVIDIA RTX 4090, 24 GB VRAM). The proposed method requires slightly higher training time due to the dual-window and texture-fusion modules.

Model	Params (M)	GPU Memory (GB)	Total Training Time (h)
ResNet50 (Baseline)	25.6	8.7	3.0
DenseNet121 (Baseline)	8.0	7.5	3.5
ConvNeXtV2 (Base)	29.0	9.2	4.0
**Ours (ConvNeXtV2 + ADWE + MTPF)**	**31.5**	**10.4**	**4.8**

As shown in Table 7, the proposed model exhibits a moderate increase in computational cost compared to the baseline networks, which is primarily attributed to the additional dual-window enhancement and texture-fusion operations. Despite this increase, the overall training efficiency remains within a practical range, with only a slight rise in GPU memory usage and total training time relative to ConvNeXtV2. This balance between computational demand and improved feature representation demonstrates that the proposed architecture achieves enhanced performance without imposing excessive resource overhead, maintaining suitability for standard deep learning hardware environments.

## 7 Conclusion

This paper addresses the challenges of insufficient contrast, blurred tissue boundaries, and complex texture variations in kidney CT image classification, and proposes a novel framework that combines Adaptive Dual-Window Enhancement with Multi-Scale Texture Prior Fusion. The ADWE module dynamically adjusts window width and window level parameters to generate complementary views, significantly enhancing the model’s discriminative ability for both soft tissues and high-density structures; the MTPF module integrates edge, local binary pattern, and Gabor features to effectively capture fine-grained texture and morphological differences. Experimental results demonstrate that the proposed method outperforms existing mainstream deep learning and domain-specific medical models in both binary and four-class classification tasks, while maintaining stable robustness under high-noise conditions, thereby validating its effectiveness and clinical value.

Future research will be further extended in three directions: first, exploring the application of multi-window enhancement and texture prior mechanisms to three-dimensional CT or multimodal imaging to improve spatial modeling of complex lesions; second, integrating self-supervised and few-shot learning approaches to further alleviate the issue of limited annotated medical imaging data; and third, introducing stronger interpretability mechanisms to make the model’s decision-making process more transparent and clinically accessible. Through these extensions and improvements, the proposed method is expected to play a broader role in intelligent diagnosis of medical imaging.

## Ethics statement

Ethical approval was not required for this study as it does not involve human participants or animals.

## References

[1] Ahmad IS, Dai J, Xie Y, Liang X. Deep learning models for CT image classification: A comprehensive literature review. Quant Imaging Med Surg. 2025;15(1):962–1011. doi: 10.21037/qims-24-1400 39838987 PMC11744119

[2] Gupta K, Bajaj V. Deep learning models-based CT-scan image classification for automated screening of COVID-19. Biomed Signal Process Control. 2023;80:104268. doi: 10.1016/j.bspc.2022.104268 36267466 PMC9556167

[3] Kalidindi A, Kompalli P, Bandi S, Anugu S. CT image classification of human brain using deep learning. 2021.

[4] Goceri E. Automatic kidney segmentation using Gaussian mixture model on MRI sequences. In: Electrical power systems and computers: Selected papers from the 2011 international conference on electric and electronics (EEIC 2011) in Nanchang, China on June 20–22, 2011, Volume 3. Springer; 2011. p. 23–9.

[5] Goceri N, Goceri E. A neural network based kidney segmentation from MR images. In: 2015 IEEE 14th international conference on machine learning and applications (ICMLA); 2015. p. 1195–8. 10.1109/icmla.2015.229

[6] Aggarwal P, Mishra NK, Fatimah B, Singh P, Gupta A, Joshi SD. COVID-19 image classification using deep learning: Advances, challenges and opportunities. Comput Biol Med. 2022;144:105350. doi: 10.1016/j.compbiomed.2022.105350 35305501 PMC8890789

[7] Wang W, Liang D, Chen Q, Iwamoto Y, Han XH, Zhang Q, et al. Medical image classification using deep learning. In: Deep learning in healthcare: Paradigms and applications. Springer; 2019. p. 33–51.

[8] Nakach F-Z, Idri A, Goceri E. A comprehensive investigation of multimodal deep learning fusion strategies for breast cancer classification. Artif Intell Rev. 2024;57(12). doi: 10.1007/s10462-024-10984-z

[9] Göçeri E. Convolutional neural network based desktop applications to classify dermatological diseases. In: 2020 IEEE 4th international conference on image processing, applications and systems (IPAS); 2020. p. 138–43.

[10] Goceri E, Karakas AA. Comparative evaluations of CNN based networks for skin lesion classification. In: 14th International conference on computer graphics, visualization, computer vision and image processing (CGVCVIP), Zagreb, Croatia; 2020. p. 1–6.

[11] Idlahcen F, Idri A, Goceri E. Exploring data mining and machine learning in gynecologic oncology. Artif Intell Rev. 2024;57(2). doi: 10.1007/s10462-023-10666-2

[12] Roth HR, Lee CT, Shin H-C, Seff A, Kim L, Yao J, et al. Anatomy-specific classification of medical images using deep convolutional nets. In: 2015 IEEE 12th international symposium on biomedical imaging (ISBI); 2015. p. 101–4. 10.1109/isbi.2015.7163826

[13] Chen X, Wang X, Zhang K, Fung K-M, Thai TC, Moore K, et al. Recent advances and clinical applications of deep learning in medical image analysis. Med Image Anal. 2022;79:102444. doi: 10.1016/j.media.2022.102444 35472844 PMC9156578

[14] Rana M, Bhushan M. Machine learning and deep learning approach for medical image analysis: Diagnosis to detection. Multimed Tools Appl. 2022:1–39. doi: 10.1007/s11042-022-14305-w 36588765 PMC9788870

[15] Sharma K, Uddin Z, Wadal A, Gupta D. Hybrid deep learning framework for classification of kidney CT images: Diagnosis of stones, cysts, and tumors. arXiv preprint; 2025. 10.48550/arXiv.250204367

[16] Maçin G, Genç F, Taşcı B, Dogan S, Tuncer T. KidneyNeXt: A lightweight convolutional neural network for multi-class renal tumor classification in computed tomography imaging. J Clin Med. 2025;14(14):4929. doi: 10.3390/jcm14144929 40725621 PMC12295850

[17] Özbay E, Özbay FA, Gharehchopogh FS. Kidney tumor classification on CT images using self-supervised learning. Comput Biol Med. 2024;176:108554. doi: 10.1016/j.compbiomed.2024.108554 38744013

[18] Bingol H, Yildirim M, Yildirim K, Alatas B. Automatic classification of kidney CT images with relief based novel hybrid deep model. PeerJ Comput Sci. 2023;9:e1717. doi: 10.7717/peerj-cs.1717 38077564 PMC10703024

[19] Zhang M, Ye Z, Yuan E, Lv X, Zhang Y, Tan Y, et al. Imaging-based deep learning in kidney diseases: Recent progress and future prospects. Insights Imaging. 2024;15(1):50. doi: 10.1186/s13244-024-01636-5 38360904 PMC10869329

[20] Zhang Y, Chen M, Zhang Z. Interpretable auto window setting for deep-learning-based CT analysis. arXiv preprint; 2025. doi: https://doi.org/arXiv:250106223 10.1016/j.compbiomed.2025.110994 40886644

[21] Lee H, Kim M, Do S. Practical window setting optimization for medical image deep learning. arXiv preprint; 2018. doi: 10.48550/arXiv.181200572

[22] Karki M, Cho J, Lee E, Hahm M-H, Yoon S-Y, Kim M, et al. CT window trainable neural network for improving intracranial hemorrhage detection by combining multiple settings. Artif Intell Med. 2020;106:101850. doi: 10.1016/j.artmed.2020.101850 32593388

[23] Mandell JC, Khurana B, Folio LR, Hyun H, Smith SE, Dunne RM, et al. Clinical applications of a CT window blending algorithm: RADIO (relative attenuation-dependent image overlay). J Digit Imaging. 2017;30(3):358–68. doi: 10.1007/s10278-017-9941-1 28097498 PMC5422232

[24] Okada N, Inoue S, Liu C, Mitarai S, Nakagawa S, Matsuzawa Y, et al. Unified total body CT image with multiple organ specific windowings: Validating improved diagnostic accuracy and speed in trauma cases. Sci Rep. 2025;15(1):5654. doi: 10.1038/s41598-024-83346-y 39955327 PMC11830084

[25] Angkurawaranon S, Sanorsieng N, Unsrisong K, Inkeaw P, Sripan P, Khumrin P, et al. A comparison of performance between a deep learning model with residents for localization and classification of intracranial hemorrhage. Sci Rep. 2023;13(1):9975. doi: 10.1038/s41598-023-37114-z 37340038 PMC10282020

[26] Inkeaw P, Angkurawaranon S, Khumrin P, Inmutto N, Traisathit P, Chaijaruwanich J, et al. Automatic hemorrhage segmentation on head CT scan for traumatic brain injury using 3D deep learning model. Comput Biol Med. 2022;146:105530. doi: 10.1016/j.compbiomed.2022.105530 35460962

[27] Songsaeng D, Supratak A, Chantangphol P, Sarumpakul S, Kaothanthong N. HU to RGB transformation with automatic windows selection for intracranial hemorrhage classification using ncCT. PLoS One. 2025;20(8):e0327871. doi: 10.1371/journal.pone.0327871 40768405 PMC12327671

[28] Dar G, Goldberg SN, Levy S, Nevo A, Daud M, Sosna J, et al. Optimal CT windowing on low-monoenergetic images using a simplex algorithm-based approach for abdominal inflammatory processes. Eur J Radiol. 2024;170:111262. doi: 10.1016/j.ejrad.2023.111262 38141262

[29] Lin X, Wang J, Wang Q, Yang Q, Li Y. Multi-window uncertainty-guided network for lung nodule CT segmentation. Alexandria Eng J. 2025;123:157–69. doi: 10.1016/j.aej.2025.03.021

[30] Li X, Xu F, Liu F, Lyu X, Gao H, Zhou J, et al. A Euclidean affinity-augmented hyperbolic neural network for semantic segmentation of remote sensing images. IEEE Trans Geosci Remote Sensing. 2025;63:1–18. doi: 10.1109/tgrs.2025.3594760

[31] Li X, Xu F, Zhang J, Yu A, Lyu X, Gao H, et al. Dual-domain decoupled fusion network for semantic segmentation of remote sensing images. Inform Fusion. 2025;124:103359. doi: 10.1016/j.inffus.2025.103359

[32] Li X, Xu F, Yu A, Lyu X, Gao H, Zhou J. A frequency decoupling network for semantic segmentation of remote sensing images. IEEE Trans Geosci Remote Sensing. 2025;63:1–21. doi: 10.1109/tgrs.2025.3531879

[33] Tong Y, Chai J, Chen Z, Zhou Z, Hu Y, Li X, et al. Dynamic frequency-decoupled refinement network for polyp segmentation. Bioengineering (Basel). 2025;12(3):277. doi: 10.3390/bioengineering12030277 40150740 PMC11939780

[34] Tong Y, Chen Z, Zhou Z, Hu Y, Li X, Qiao X. An edge-enhanced network for polyp segmentation. Bioengineering (Basel). 2024;11(10):959. doi: 10.3390/bioengineering11100959 39451335 PMC11504364

[35] Gao Y, Tan J, Shi Y, Zhang H, Lu S, Gupta A, et al. Machine learned texture prior from full-dose CT database via multi-modality feature selection for Bayesian reconstruction of low-dose CT. IEEE Trans Med Imaging. 2023;42(11):3129–39. doi: 10.1109/TMI.2021.3139533 34968178 PMC9243192

[36] Varghese BA, Fields BKK, Hwang DH, Duddalwar VA, Matcuk GR Jr, Cen SY. Spatial assessments in texture analysis: What the radiologist needs to know. Front Radiol. 2023;3:1240544. doi: 10.3389/fradi.2023.1240544 37693924 PMC10484588

[37] Xu W, Shi X. Integrating radiomic texture analysis and deep learning for automated myocardial infarction detection in cine-MRI. Sci Rep. 2025;15(1):24365. doi: 10.1038/s41598-025-08127-7 40628813 PMC12238615

[38] Wang Q, Chen Y, Zhang N, Gu Y. Medical image inpainting with edge and structure priors. Measurement. 2021;185:110027. doi: 10.1016/j.measurement.2021.110027

[39] Corrias G, Micheletti G, Barberini L, Suri JS, Saba L. Texture analysis imaging “what a clinical radiologist needs to know”. Eur J Radiol. 2022;146:110055. doi: 10.1016/j.ejrad.2021.110055 34902669

[40] Materka A. Texture analysis methodologies for magnetic resonance imaging. Dialogues Clin Neurosci. 2004;6(2):243–50. doi: 10.31887/DCNS.2004.6.2/amaterka 22033841 PMC3181797

[41] van Timmeren JE, Cester D, Tanadini-Lang S, Alkadhi H, Baessler B. Radiomics in medical imaging-"how-to" guide and critical reflection. Insights Imaging. 2020;11(1):91. doi: 10.1186/s13244-020-00887-2 32785796 PMC7423816

[42] Chai S, Rueckert D, Fetit AE. Reducing textural bias improves robustness of deep segmentation models. In: Annual conference on medical image understanding and analysis. Springer; 2021. p. 294–304.

[43] Fekri-Ershad S. A review on image texture analysis methods. arXiv preprint arXiv:180400494; 2018.

[44] Simonyan K, Zisserman A. Very deep convolutional networks for large-scale image recognition. arXiv preprint; 2014. https://arxiv.org/abs/1409.1556

[45] He K, Zhang X, Ren S, Sun J. Deep residual learning for image recognition. In: 2016 IEEE conference on computer vision and pattern recognition (CVPR); 2016. p. 770–8. 10.1109/cvpr.2016.90

[46] Xie S, Girshick R, Dollár P, Tu Z, He K. Aggregated residual transformations for deep neural networks. In: Proceedings of the IEEE conference on computer vision and pattern recognition; 2017. p. 1492–1500.

[47] Liu Z, Mao H, Wu CY, Feichtenhofer C, Darrell T, Xie S. A convnet for the 2020 s. In: Proceedings of the IEEE/CVF conference on computer vision and pattern recognition; 2022. p. 11976–86.

[48] Dosovitskiy A, Beyer L, Kolesnikov A, Weissenborn D, Zhai X, Unterthiner T, et al. An image is worth 16x16 words: Transformers for image recognition at scale. arXiv preprint arXiv:201011929; 2020.

[49] Liu Z, Lin Y, Cao Y, Hu H, Wei Y, Zhang Z. Swin transformer: Hierarchical vision transformer using shifted windows. In: Proceedings of the IEEE/CVF international conference on computer vision; 2021. p. 10012–22.

[50] Huo X, Sun G, Tian S, Wang Y, Yu L, Long J, et al. HiFuse: Hierarchical multi-scale feature fusion network for medical image classification. Biomed Signal Process Control. 2024;87:105534. doi: 10.1016/j.bspc.2023.105534

[51] Han Q, Qian X, Xu H, Wu K, Meng L, Qiu Z, et al. DM-CNN: Dynamic multi-scale convolutional neural network with uncertainty quantification for medical image classification. Comput Biol Med. 2024;168:107758. doi: 10.1016/j.compbiomed.2023.107758 38042102

[52] Soleimani-Fard S, Ko S. Res-MGCA-SE: A lightweight convolutional neural network based on vision transformer for medical image classification. Neural Comput Applic. 2024;36(28):17631–44. doi: 10.1007/s00521-024-10053-0

[53] Yue Y, Li Z. Medmamba: Vision mamba for medical image classification. arXiv preprint; 2024. https://doi.org/arXiv:240303849

